# Optimal piezoelectric shunt dampers for non-deterministic substructure vibration control: estimation and parametric investigation

**DOI:** 10.1038/s41598-021-84097-w

**Published:** 2021-02-25

**Authors:** Asan G. A. Muthalif, Azni N. Wahid

**Affiliations:** 1 Department of Mechanical and Industrial Engineering, College of Engineering, Qatar University, Doha, Qatar; 2 Smart Structures, Systems, and Control Research Lab, Department of Mechatronics Engineering, International Islamic University Malaysia, Selangor, Malaysia

**Keywords:** Mechanical engineering, Actuators

## Abstract

Piezoelectric (PZT) shunt damping is an effective method to dissipate energy from a vibrating structure; however, most of the applications focus on targeting specific modes for structures vibrating at low-frequency range, i.e. deterministic substructure (DS). To optimally attenuate structures vibrating at high-frequency range, i.e. non-deterministic substructure (Non-DS) using a PZT shunt damper, it is found that the impedance of the PZT patch’s terminal needs to be the complex conjugate of its inherent capacitance paralleled with the impedance ‘faced’ by its non-deterministic host structure underline moment actuation. The latter was derived in terms of estimation of the effective line moment mobility of a PZT patch on a Non-DS plate by integrating the expression of driving point moment mobility of an infinite thin plate. This paper conducts a parametric investigation to study the effect of changing the size, quantity and configuration of the PZT patch to the performance of the optimal PZT shunt dampers in dissipating the energy of its non-deterministic host structure. Results are shown in terms of energy reduction ratio of the thin plate when attached with optimal PZT shunt damper(s).

## Introduction

**Figure 1:**
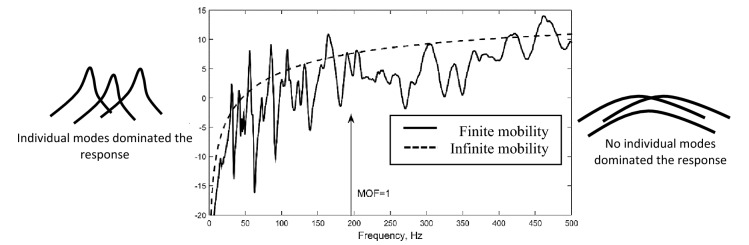
Typical driving point mobility of a finite rectangular plate with its infinite mobility serves as an approximation at the high-frequency range. MOF when unity is also shown^7^.

Substructures which experience long-wavelength deformation are called deterministic (DS) and their response can be described mathematically, while non-deterministic substructures (Non-DS) experience short-wavelength deformation and very sensitive to structural uncertainties which makes it hard to be modelled. In Statistical Energy Analysis (SEA), modal overlapping factor (MOF) is used to categorize the Non-DS and DS; it is essentially the average number of resonances that fall within the half power bandwidth of a single resonance (degree of modal overlap). The expression is^1–3^:

1 $$MOF\left(\omega \right)=n\omega \eta = \frac{A}{4\pi }\sqrt{\frac{\rho h}{D}}\omega \eta ,$$

where $\omega$ in rad/s, *n* and $\eta$ refers to modal density and loss factor, respectively. Surface area, density, thickness and flexural rigidity are denoted as *A,* $\rho ,h$ and *D,* respectively. The response at MOF < 1 exhibit distinctly visible modal responses which can be simulated using the finite element method (FEM). Response that lies at 1 < MOF < 2 is categorized as mid-frequency range and MOF > 2 is categorized as high-frequency range. At high frequency range, small structural uncertainties can cause a big difference in the vibration response^1,4,5^ and modal response does not exhibit individual resonant but rather broader peaks^6^ (Fig. 1) which makes it hard to be controlled. In addition to that, higher computational time and cost is needed to capture the short-wavelength response at higher frequency region. Physically, short wavelength vibration can transmit through tiny structural cracks which eventually causes failure. Hence, it is essential to be able to model high frequency in order to control vibration of Non-DS.

Various works on high-frequency vibration analysis focused on finding the best model to predict the dynamic properties of high-frequency vibration to understand its behavior. However, there are not many research done based on high-frequency vibration control which specify the range of frequency to be controlled in line with SEA context i.e. frequency with respect to MOF. One of the methods to analyze high-frequency vibration response is by using the Hybrid Modelling method^8^ which combines deterministic modelling approach (FEM) with statistical modelling method (SEA), where components in complex built-up structures are grouped into DS and Non-DS, and these are coupled together to calculate the response for the whole structure.

An attempt to develop active control for structures vibrating at the high-frequency range has been discussed in work by Muthalif^6^ where they used a skyhook damper (equivalent to point force) to dissipate energy from a non-deterministic thin plate. The optimal value for the skyhook damper constant is achieved by doing the Hybrid Modelling equation’s first derivative. The work has proved that ‘impedance matching’ method is valid for a successful energy dissipation from a Non-DS that is by making the impedance of the skyhook damper to be the complex conjugate of the impedance ‘seen’ by the non-deterministic plate at the point junction. As an extension of the work, this paper will attempt to dissipate energy from a thin plate vibrating at high-frequency range in a passive manner since the traditional passive control has better control effect in dealing with high-frequency vibration^9–12^. Compared to active feedback control, PZT shunt damping is more attractive in terms of being entirely passive without a power supply requirement, ensuring stability and therefore more practical to be implemented in the field^11,13^. The majority of shunt circuit configurations also do not require a parametric model of the plant; therefore, it is more convenient to design and tune^10,14^.

It is intriguing to know the effect of changing certain physical parameters of the PZT patch to the optimal PZT shunt dampers’ performance in dissipating the energy of its non-deterministic host structure. This paper investigates the effect of changing the size, quantity, and configuration of the PZT patch, respectively, in reducing its non-deterministic thin plate’s energy. Firstly, the PZT shunt damper’s optimal circuit impedance to maximize energy dissipation from its non-deterministic host structure (a thin plate is used) needs to be derived. For that purpose, the dynamic effect of a PZT patch on a non-deterministic thin plate needs to be known due to its electromechanical properties and having different forcing distributions compared to a skyhook damper which is a straightforward point force mechanical damper. The approach taken here is to model a PZT patch transducer as line moment exciter on a randomized thin plate and to estimate its mobility function using infinite mobility term.

This article is organized as follows; “Derivation of optimal impedance for non-DS control” section shows the mathematical derivation to obtain the optimal impedance to achieve maximum energy dissipation of a Non-DS attached with a PZT shunt damper by utilizing the Hybrid Modelling method equation. “Dynamic electromechanical response of a piezoelectric shunt damper on a randomized thin plate” section shows the dynamic electromechanical response of a randomized thin plate attached with a PZT shunt damper. “Derivation of effective line moment mobility on infinite thin plate” section shows the derivation for the estimation of effective line moment mobility to be used in the theoretical optimal shunt impedance equation derived in “Derivation of optimal impedance for non-DS control” section. “Simulation studies on parametric investigations” section compiles simulation studies on changing the size, quantity, and connection of the *optimal* PZT shunt dampers, respectively, to the energy reduction ratio achieved by the non-deterministic thin plate.

## Derivation of optimal impedance for Non-DS control

**Figure 2:**
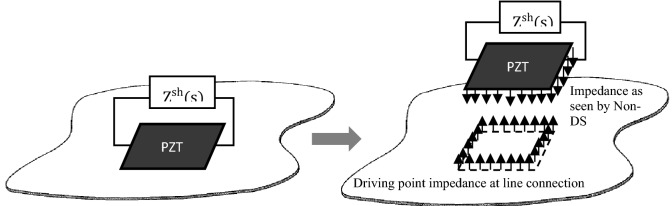
A PZT patch with a shunt circuit, ${\mathrm{Z}}^{\mathrm{sh}}(\mathrm{s})$ acting on a non-deterministic plate. The patch can be regarded as line moment exciter on the Non-DS, therefore the driving point impedance at line connection needs to be established^7^.

In this section, the hybrid FEA-SEA^6,8^ is utilized to derive the optimal impedance for Non-DS control. The reason is that its modelling approach treats a complex built-up system as a combination of components with fully deterministic properties (DS) and substructure that have a high degree of randomness (Non-DS)^8,15^. Consider the simplest form of a built-up structure where a shunted PZT patch is directly attached on a randomized thin plate as shown in Fig. 2; the patch with its circuit is treated as a DS and the host substructure as a Non-DS. The strategy for Non-DS vibration suppression is pursued by finding the DS’s optimal impedance when the energy loss at the DS is maximized, using the first derivative of the hybrid (FE/SEA) method^6,16^.

The equation used to find the Non-DS energy (SEA part of the hybrid method) for the system in Fig. 2 is^8^:

2 $$\left(\omega {\eta }_{d,1}{n}_{1}+\omega {\eta }_{1}{n}_{1}\right)\frac{{E}_{1}}{{n}_{1}}={P}_{in}.$$

where ${\eta }_{1}$ and ${n}_{1}$ are the loss factor and modal density of the Non-DS,${\eta }_{d,1}$ is the loss factor of the DS (the controller), $\frac{{E}_{1}}{{n}_{1}}$ is the modal energy of the subsystem and ${P}_{in}$ is the power input to the subsystem. If the power input to the Non-DS is fixed, then increasing the magnitude of $\omega {\eta }_{d,1}{n}_{1}$ will then decrease the energy of the Non-DS, $\frac{{E}_{1}}{{n}_{1}}(\omega {\eta }_{1}{n}_{1})$ which fulfils the control purpose. The first term in Eq. (2) is given by the hybrid method as^8^:

3 $$\omega {\eta }_{d,k}{n}_{k}=\frac{2}{\pi }\sum_{rs}Im\left({{\varvec{D}}}_{d,rs}\right){({{\varvec{D}}}_{tot}^{-1}Im\{{{\varvec{D}}}_{dir}^{k}\}{{\varvec{D}}}_{tot}^{-H})}_{rs},$$

where ${{\varvec{D}}}_{d,rs}$ is the complex dynamic stiffness matrix for the DS, ${{\varvec{D}}}_{dir}^{k}$ is the complex direct dynamic stiffness matrix at the coupling points (line or area), ${{\varvec{D}}}_{tot}$ is the sum of ${{\varvec{D}}}_{d,rs}$ and ${{\varvec{D}}}_{dir}^{k}$, ${{\varvec{D}}}_{tot}^{-H}$ is the inverse of hermitian transpose for ${{\varvec{D}}}_{tot}$. The relationship between complex dynamic stiffness to structural impedance is ${D}_{d,DS}=j\omega {Z}_{d}$ and ${D}_{dir}=j\omega {Z}_{\infty }$ where ${Z}_{D}$ is the impedance of the DS as “seen” by the Non-DS and ${Z}_{\infty }$ is the infinite plate driving point impedance at the connection between the DS and Non-DS. While this is unambiguous for a skyhook damper which is a mechanical damper with point junction, the impedance expression needs a more precise definition for other types of controller; in this case, a PZT shunt damper is an electromechanical transducer and has completely different forcing distribution and spatial connection with its host structure.

Consider *N* number of PZT shunt dampers attached on the Non-DS, the optimal impedance value for each of the DS to maximize the value of energy loss, ${\eta }_{d,1}$ is obtained by doing the first derivative of Eq. (3) concerning both the real part and the imaginary part of ${D}_{d,k}$ for the *k*^th^ DS separately, will lead to:

4 $${D}_{dR,k}=-{D}_{dirR,k},$$

5 $${D}_{dI,k}={D}_{dirI,k}.$$

Equations (4) and (5) are the optimal impedance for a DS or specifically, the optimal impedance of the *k*th shunted PZT patch needed to minimize the Non-DS’s energy which is essentially equivalent to the well-known ‘impedance matching technique’. The equations also illustrate that the optimal gain value for deterministic controllers is independently related to the direct dynamic stiffness of its non-deterministic host structure at their respective point/line/area connections. This finding will significantly simplify the controller’s design. Evidently, knowing the mobility function of a structure is crucial to determine the optimal controller design for maximum energy dissipation from a Non-DS.

Further investigation using the above derivation also reveals that the theoretical energy ratio between a bare plate and a controlled plate using optimized DS, hereby defined as ${E}_{ratio, Inf}$ becomes^6^:

6 $${E}_{ratio, Inf}=\frac{{E}_{o}}{{E}_{c}}=1+\left(\frac{N}{2\pi \omega {\eta }_{o}n}\right),$$

where *N* is the quantity of the optimized controller; in this case, it is the PZT patch with its terminal connected to an optimal shunt impedance. As the frequency is made higher, the energy ratio between a bare plate, *E*_*o*_ and a controlled plate, ${E}_{c}$, will approach to unity, $\frac{{E}_{o}}{{E}_{c}}=1;$ which implies that control for a Non-DS is ineffective at very high frequency. However, the number of controllers, *N* can be increased to alleviate this drawback.

### The optimal shunt circuit for piezoelectric shunt damper

**Figure 3:**
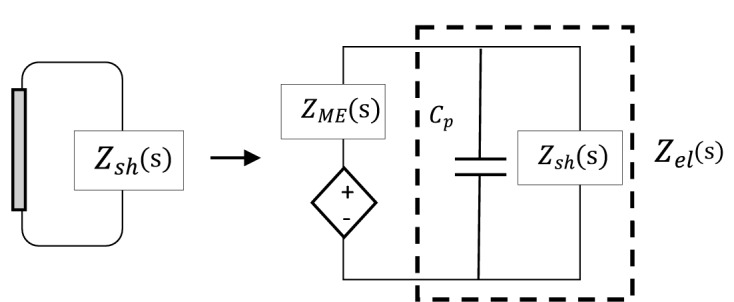
Physical model of a shunted PZT patch and its equivalent network analog.

Figure 3 shows the physical model of a PZT shunt damper with circuit impedance ${Z}_{sh}(\mathrm{s})$ and its equivalent electrical network representation. ${Z}_{ME}$ is the mechanical impedance of the patch (its mass and stiffness), ${C}_{p}$ is the patch’s inherent capacitance, and ${Z}_{el}$ is the electrical representation of the PZT shunt damper, that is the electrical shunt impedance parallel with inherent capacitance impedance of the patch i.e. ${Z}_{sh}||{Z}_{Cp}$ or:

7 $${Z}_{el}=\frac{{Z}_{sh}{Z}_{Cp}}{{Z}_{sh}+{Z}_{Cp}}=\frac{{jZ}_{sh}{X}_{Cp}}{{Z}_{sh}+{jX}_{Cp}},$$

where ${X}_{Cp}=-\frac{1}{\omega {C}_{p}}.$ Since the PZT patch is attached to a non-deterministic thin plate which is equivalent to an infinite thin plate^17^, the mass and stiffness of the patch can be neglected; therefore the DS shown in Fig. 3 consists only the patch’s inherent capacitance with its shunt circuit i.e. ${Z}_{el}\left(\mathrm{s}\right).$ The *mechanical* dynamic stiffness matrix of the deterministic part of the system can be written as^18^:

8 $$D_{d} = j\omega \Gamma^{T} \varphi_{{M_{line} }}^{ - 1} Z_{el} \varphi_{{\dot{\theta }_{line,1} }}^{ - 1} \Gamma .$$

Substituting Eqs. (8) into (7), and using conditions in Eqs. (4) and (5) to obtain maximum energy dissipation at the deterministic controller; the optimal shunt circuit, ${Z}_{sh}$ to be designed can be expressed in terms of complex direct dynamic stiffness, $\overline{D}_{dir} = \overline{D}_{Redir} + j\overline{D}_{Imdir}$, where the following expression is obtained:

9 $$Z_{sh} = \frac{{\omega X_{Cp}^{2} \overline{D}_{Imdir} }}{{\overline{D}_{Imdir}^{2} + ( - \overline{D}_{Redir} - \omega X_{Cp} )^{2} }} - j\frac{{\overline{D}_{Imdir}^{2} X_{Cp} + X_{Cp} \overline{D}_{Redir} \left( {\overline{D}_{Redir} + \omega X_{Cp} } \right)}}{{\overline{D}_{Imdir}^{2} + ( - \overline{D}_{Redir} - \omega X_{Cp} )^{2} }}.$$

The bar sign indirect dynamic stiffness term, $\overline{D}_{dir}$ signifies the equivalent electrical dynamic stiffness matrix, converted from its mechanical dynamic stiffness matrix, $D_{dir}$ using some conversion factor:

10 $$\overline{D}_{dir} = \varphi_{{M_{line} }} \Gamma_{{}}^{ - T} D_{dir} \Gamma_{{}}^{ - 1} \varphi_{{\dot{\theta }_{line} }} ,$$

where the terms $\varphi_{{M_{line,k} }} { }$ and $\varphi_{{\dot{\theta }_{line,k} }}$ account for the spatial coupling factor of the PZT patch on the thin plate and $\Gamma_{{}}^{ - 1}$ accounts for electromechanical coupling for conversion from mechanical to an electrical parameter. Rewriting Eq. (9) in terms of infinite mechanical impedance, $Z_{\infty }$. will produce the following expressions:

11 $$Z_{sh} = \frac{{X_{Cp}^{2} \overline{Z}_{Re\infty } }}{{\overline{Z}_{Re\infty }^{2} + (\overline{Z}_{Im\infty } + X_{Cp} )^{2} }} - j\frac{{\overline{Z}_{Re\infty }^{2} X_{Cp} + X_{Cp} \overline{Z}_{Im\infty } \left( {\overline{Z}_{Im\infty } + X_{Cp} } \right)}}{{\overline{Z}_{Re\infty }^{2} + (\overline{Z}_{Im\infty } + X_{Cp} )^{2} }},$$

where similarly, the bar sign indicates conversion to electrical parameter through some factor:

12 $$\overline{Z}_{\infty } = \varphi_{{M_{line} }} \Gamma^{ - T} Z_{\infty } \Gamma^{ - 1} \varphi_{{\dot{\theta }_{line} }} .$$

Equation (11) is, therefore, the optimal shunt impedance for the PZT shunt damper attached on its non-deterministic host structure to maximize energy dissipation; which is the complex conjugate of its inherent capacitance, $X_{Cp}$ paralleled with the mechanical-converted-to-electrical impedance ‘faced’ by the Non-DS at the junction, represented by the term $Z_{\infty }$. The derivation of $Z_{\infty }$ will be shown in “Derivation of effective line moment mobility on infinite thin plate” section using *effective line moment mobility* concept.

## Dynamic electromechanical response of a piezoelectric shunt damper on a randomized thin plate

Equation of motion of a thin plate with a PZT patch shunt damper is derived in this section with reference from^19^. Hagood et al. showed how to use the constitutive equation for PZT material to obtain the general equation for a PZT in terms of the external current input and applied voltage. Rearranging the terms, the following is obtained:

13 $$\left[ {\begin{array}{*{20}c} I \\ {\sigma_{pzt} } \\ \end{array} } \right] = \left[ {\begin{array}{*{20}c} {Y^{EL} } & {sAe} \\ { - e^{t} t_{p}^{ - 1} } & {c^{E} } \\ \end{array} } \right]\left[ {\begin{array}{*{20}c} V \\ S \\ \end{array} } \right],$$

14 $$Y^{EL} = Y_{PZT}^{D} + Y^{sh} ,$$

where *I* is the electric current,$\sigma_{pzt}$ is stress vector for the PZT patch, $c^{E}$ is the modulus of elasticity of the PZT patch, *e* is PZT coupling coefficient matrix and its transpose, $e^{t} .$ *V* is the electric voltage, *S* is the strain, *A* is the surface area perpendicular to the electrical field, $t_{p}$ is the thickness of the patch, $Y^{EL}$ is the electrical admittance, $Y^{sh}$ is electrical admittance of the shunt circuit, $Y_{PZT}^{D} = sC_{p}^{s}$ where $C_{p}^{s}$ is the inherent capacitance of the patch at constant strain and *s* is Laplace domain function. The stress expression is updated as:

15 $$\sigma_{pzt} = [c^{E} + e^{t} \overline{Z}^{EL} \left( {\varepsilon^{S} } \right)^{ - 1} e\left] {S - } \right[e^{t} t_{p}^{ - 1} Z^{EL} ]I.$$

And the new modulus of elasticity for a shunted PZT patch, $c^{shunt}$ is defined as:

16 $$c^{shunt} = \left[ {c^{E} + e^{t} \overline{Z}^{EL} \left( {\varepsilon^{S} } \right)^{ - 1} e} \right],$$

where the matrix of non-dimensional electrical impedance, $\overline{Z}^{EL}$ is:

17 $$\overline{Z}^{EL} = Z^{EL} \left( {Z_{PZT}^{D} } \right)^{ - 1} = \left( {sC_{p}^{s} + Y^{sh} } \right)^{ - 1} sC_{p}^{s} ,$$

$\overline{Z}^{EL} = 1$ is for open circuit condition.

Hence, the total equation of motion for a thin plate attached with a PZT patch connected to a shunt circuit, $$Z^{sh} \left( s \right)$$ where the impedance is inverse of admittance/mobility ($$Z^{u} = 1/Y^{u} )$$ becomes:

18 $$- \omega^{2} \left( {M_{plate} + M_{pzt} + M_{ptmass} } \right)W_{mn,s} + \left( {K_{plate} + K_{pzt} + \Gamma^{T} \overline{Z}^{EL} \varepsilon^{S - 1} \Gamma } \right)W_{mn,s} = \Gamma Z^{EL} I\left( \omega \right) + \phi_{f} F_{i} \left( \omega \right),$$

where $M_{plate} , M_{pzt}$ and $M_{ptmass}$ are the masses of the thin plate, patch and the distributed point masses for randomization, respectively. $K_{plate}$ and $K_{pzt}$ are the stiffness matrices of the plate and PZT patch, respectively. $I\left( \omega \right)$ and $F_{i} \left( \omega \right)$ are the current input to the shunt circuit and force input to the system, respectively, $\Gamma_{{}}$ is the electromechanical coupling matrix and its transpose, $\Gamma^{T} ,$ and $\varepsilon^{S}$ is the dielectric permittivity at constant strain. Solving for modal coordinate, $W_{mn,s}$ yields:

19 $$W_{mn,s} = \Delta_{s}^{ - 1} \Gamma Z^{EL} I\left( \omega \right) + \Delta_{s}^{ - 1} \phi_{f} F_{i} \left( \omega \right),$$

where $\Delta_{s} \left( {\upomega } \right)$ is expressed as:

20 $$\Delta_{s} \left( \omega \right) = - \omega^{2} \left( {M_{plate} + M_{pzt} + M_{ptmass} } \right) + \left( {K_{plate} + K_{pzt} + \Gamma^{T} \overline{Z}^{EL} \varepsilon^{S - 1} \Gamma } \right).$$

The finite energy ratio between a thin plate attached with an open-circuited PZT shunt damper and a controlled plate with closed-circuited PZT shunt damper can be written as:

21 $$E_{ratio, fin} = \frac{{E_{o,fin} }}{{E_{c,fin} }},$$

where

22 $$E_{o,fin} = \frac{1}{2}W_{mn,o}^{T} K_{plate} W_{mn,o} , {\text{where}} \overline{Z}^{EL} = 1 ,$$

for open circuit terminal and:

23 $$E_{c,fin} = \frac{1}{2}W_{mn,s}^{T} K_{plate} W_{mn,s} , {\text{where}} \overline{Z}^{EL} = \left( {sC_{p}^{s} + Y^{sh} } \right)^{ - 1} sC_{p}^{s} ,$$

when a shunt circuit with admittance $Y^{sh}$ is connected to the terminal. Equation (21)’s finite energy ratio will be compared with its theoretical expression using the infinite model as derived in Eq. (6) for validation.

## Derivation of effective line moment mobility on infinite thin plate

Power transmission between the contract region of source and receiver is better approximated using surface mobilities. Traditionally, point-like connection with connection area of less than 1/10 of wavelength is assumed between isolator and main structure^20,21^. However, for a PZT patch transducer, dimension of the connection area is comparable to the wavelength. The concept of strip mobility is introduced by Hammer and Petersson to study power transmission to a thin plate^22^. Subsequently, surface mobility concept on a circular contact area has been developed by Norwood et al.^20^. Li et al.^23^ and Dai et al.^24^ extended the work for square-shaped area to find surface mobility using discritized model.

### Infinite mobilities model

A multi-point connection model is employed in this article, where the infinite thin plate mobility subjected to PZT induced line moment at edges^25^ is obtained through the integration method. This is termed as *effective line moment mobility* and requires prior derivation of the *effective point moment* mobility. Ljunggeren^26^ regarded force applied along with an infinite line as infinite point forces and thus derived effective point mobility from point excited fields. Using the same principle, effective point moment mobility produced by a *finite* line moment can be obtained.

**Figure 4:**
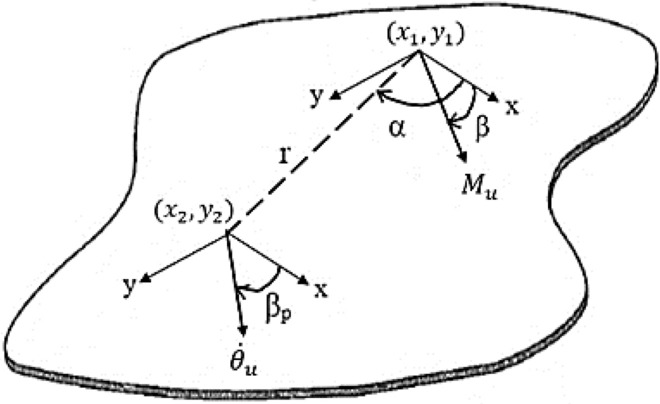
Illustrations of sign conventions for resulting angular velocity at point (${\text{x}}_{2}$,${\text{ y}}_{2}$) subjected by a point moment excitation at (${\text{x}}_{1}$,${\text{ y}}_{1}$)^7^.

The angular displacement at (*r*,α), in response to a couple of *point* moment $M_{u}$ with orientation *u* which acts on a rigid indenter fixed to the plate, is given as (see Fig. 4)^21^:

24 $$\begin{aligned} \theta_{u}^{^{\prime}} \left( {r,\alpha } \right) &= \left( {\frac{{M_{u} }}{j\omega }} \right)\frac{\omega }{8D}\left\{\vphantom{{+ \frac{{\cos \left( {\alpha - \beta_{p} } \right)\cos \left( {\alpha - \beta } \right)}}{{k_{B} r}}\left( {H_{1}^{\left( 2 \right)} \left( {k_{B} r} \right) - j\frac{2}{\pi }K_{1} \left( {k_{B} r} \right)} \right)}} {\sin \left( {\alpha - \beta_{p} } \right)\sin \left( {\alpha - \beta } \right)\left[ {\left( {H_{0}^{\left( 2 \right)} \left( {k_{B} r} \right) - j\frac{2}{\pi }K_{0} \left( {k_{B} r} \right)} \right) - \frac{1}{{k_{B} r}}\left( {H_{1}^{\left( 2 \right)} \left( {k_{B} r} \right) - j\frac{2}{\pi }K_{1} \left( {k_{B} r} \right)} \right)} \right]}\right.\\&\quad \left.{+ \frac{{\cos \left( {\alpha - \beta_{p} } \right)\cos \left( {\alpha - \beta } \right)}}{{k_{B} r}}\left( {H_{1}^{\left( 2 \right)} \left( {k_{B} r} \right) - j\frac{2}{\pi }K_{1} \left( {k_{B} r} \right)} \right)} \right\}, \end{aligned}$$

where $H_{i}^{\left( 2 \right)} \left( {k_{B} r} \right)$ is the second kind of Hankel function of the *i-*th order, $K_{i} \left( {k_{B} r} \right)$ is the second kind of modified Bessel function of the *i-*th order, $k_{B} = {\sqrt[4]{{\frac{{\omega^{2} \rho h}}{D}}}}$ is the bending wavenumber, *r* is the distance between the force applied and velocity measured, $\beta$ is the angle between x-axis and moment arm, $M_{u}$ and $\alpha$ is the angle between the x-axis and the radius line that connects point ($x_{1}$, $y_{1}$) and ($x_{2}$, $y_{2}$). The angular displacement at a point resulting from *line* moment excitation with length *b-a* can be taken as:

25 $$\theta_{u} = \mathop \smallint \limits_{a}^{b} \theta_{u}^{^{\prime}} \left( r \right)dr,$$

where *a* and *b* are finite numbers which account for the edge coordinate of the line moment and *r* is the distance between the response point and the moment excitation along the line.

26 $$r = \sqrt {\left( {x_{2} - x_{1} } \right)^{2} + \left( {y_{2} - y_{1} } \right)^{2} } .$$

Generally, *r* can be also be written as $r = \sqrt {\left( {x_{{\dot{\theta }_{u} }} - x_{{M_{u} }} } \right)^{2} + \left( {y_{{\dot{\theta }_{u} }} - y_{{M_{u} }} } \right)^{2} }$; where $(x_{{{\dot{\theta }}_{{\text{u}}} }} ,y_{{{\dot{\theta }}_{{\text{u}}} }} )$ and $\left( {x_{{{\text{M}}_{{\text{u}}} }} ,y_{{{\text{M}}_{{\text{u}}} }} } \right)$ are the coordinates of the resulting angular velocity and the point moment, respectively. Since angular velocity is taken at one fixed point ($x_{2} , y_{2}$), then the coordinate of the point moment will be the variable to be integrated to produce line moment.

**Figure 5:**
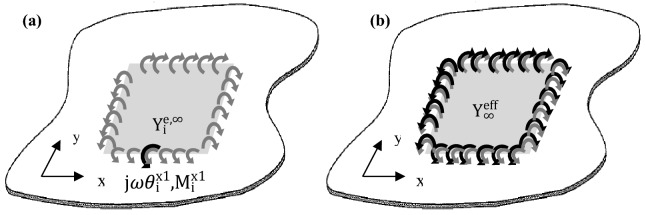
Illustration of (**a**) effective point moment mobility at one point on line x_1_ and (**b**) effective line moment mobility of the infinite thin plate^7^.

The *effective point moment* mobility, $Y_{i}^{e}$ for the infinite thin plate at a point *i* on the line moment is, therefore:

27 $$\begin{aligned} Y_{i}^{e,\infty } = \,& \frac{{j\omega \theta_{i}^{u} }}{{M_{i}^{u} }} = \frac{j\omega }{{M_{i}^{u} }}\mathop \smallint \limits_{a}^{b} \theta_{u}^{^{\prime}} \left( r \right)dr \\ =\, & \frac{j\omega }{{M_{i}^{u} }}\mathop \smallint \limits_{{x_{p1} }}^{{x_{p2} }} \theta_{u}^{^{\prime}} \left( {x_{{{\dot{\theta }}_{{\text{u}}} }} ,y_{{{\dot{\theta }}_{{\text{u}}} }} ,x_{{{\text{M}}_{{\text{u}}} }} ,y_{{{\text{M}}_{{\text{u}}} }} } \right)dx_{{{\text{M}}_{{\text{u}}} }} + \frac{j\omega }{{M_{i}^{u} }}\mathop \smallint \limits_{{y_{p1} }}^{{y_{p2} }} \theta_{u}^{^{\prime}} \left( {x_{{{\dot{\theta }}_{{\text{u}}} }} ,y_{{{\dot{\theta }}_{{\text{u}}} }} ,x_{{{\text{M}}_{{\text{u}}} }} ,y_{{{\text{M}}_{{\text{u}}} }} } \right)dy_{{{\text{M}}_{{\text{u}}} }} , \\ \end{aligned}$$

where $M_{i}^{u}$, is the excitation moment at *i*th connection point (see Fig. 5a), $\theta_{u}^{^{\prime}}$ is Eq. (23), $x_{p2}$−$x_{p1}$ are the length of the patch along x-axis and $y_{p2}$−$y_{p1}$ are the length of the patch along the y-axis. Extending the same method to PZT patch (considering pure line moments at edges) attached infinite plate, the effective point moment mobility at point $\left( {x_{{{\dot{\theta }}_{{{\text{x}}1}} }} ,y_{{{\dot{\theta }}_{{{\text{x}}1}} }} } \right)$ can be evaluated. The *effective line moment* mobility, $Y_{\infty }^{eff}$ can be obtained as a summation of $Y_{i}^{e}$ for all connection points. In this case, the connection point is assumed to be along the length of the edges of the PZT patch (Fig. 5b):

28 $$\begin{aligned} Y_{\infty }^{eff} = & \int\limits_{u} {Y_{i}^{e,\infty } du} = j\omega \mathop \smallint \limits_{u}^{{}} \mathop \smallint \limits_{a}^{b} \frac{1}{{M_{i}^{u} }}\theta_{u}^{^{\prime}} \left( r \right)drdu \\ = & \mathop \smallint \limits_{{x_{p1} }}^{{x_{p2} }} \frac{j\omega }{{M_{i}^{u} }}\left[ {\mathop \smallint \limits_{{x_{p1} }}^{{x_{p2} }} \theta_{u}^{^{\prime}} \left( {x_{{{\dot{\theta }}_{{\text{u}}} }} ,y_{{{\dot{\theta }}_{{\text{u}}} }} ,x_{{{\text{M}}_{{\text{u}}} }} ,y_{{{\text{M}}_{{\text{u}}} }} } \right)dx_{{{\text{M}}_{{\text{u}}} }} + \mathop \smallint \limits_{{y_{p1} }}^{{y_{p2} }} \theta_{u}^{^{\prime}} \left( {x_{{{\dot{\theta }}_{{\text{u}}} }} ,y_{{{\dot{\theta }}_{{\text{u}}} }} ,x_{{{\text{M}}_{{\text{u}}} }} ,y_{{{\text{M}}_{{\text{u}}} }} } \right)dy_{{{\text{M}}_{{\text{u}}} }} } \right]dx_{{{\dot{\theta }}_{{\text{u}}} }} \\ \; \quad+ \,& \mathop \smallint \limits_{{y_{p1} }}^{{y_{p2} }} \frac{j\omega }{{M_{i}^{u} }}\left[ {\mathop \smallint \limits_{{x_{p1} }}^{{x_{p2} }} \theta_{u}^{^{\prime}} \left( {x_{{{\dot{\theta }}_{{\text{u}}} }} ,y_{{{\dot{\theta }}_{{\text{u}}} }} ,x_{{{\text{M}}_{{\text{u}}} }} ,y_{{{\text{M}}_{{\text{u}}} }} } \right)dx_{{{\text{M}}_{{\text{u}}} }} + \mathop \smallint \limits_{{y_{p1} }}^{{y_{p2} }} \theta_{u}^{^{\prime}} \left( {x_{{{\dot{\theta }}_{{\text{u}}} }} ,y_{{{\dot{\theta }}_{{\text{u}}} }} ,x_{{{\text{M}}_{{\text{u}}} }} ,y_{{{\text{M}}_{{\text{u}}} }} } \right)dy_{{{\text{M}}_{{\text{u}}} }} } \right]dy_{{{\dot{\theta }}_{{\text{u}}} }} . \\ \end{aligned}$$

Essentially, $Y_{\infty }^{eff}$ is the inverse of mechanical impedance as seen by the Non-DS when excited by the line moments of the patch i.e. $Z_{\infty } = Y_{\infty }^{eff}$.The theoretical optimal shunt impedance, $Z_{sh}$ as shown in Eq. (11) can now be solved incorporating Eq. (27), to maximize energy dissipation from its non-deterministic host structure, i.e. a thin plate.

## Simulation studies on parametric investigations

To gain more understanding of the behaviour of the effective line moment mobilities, and correspondingly the performance of the optimal PZT shunt dampers, the dynamic effects of changing certain parameters of the PZT patch to the effective line moment mobility of the non-deterministic thin plate with the corresponding control effectiveness of to the resulting shunt circuit will be performed thru parametric investigations. The physical parameters investigated include (1) changing the patch dimension using 3 × 3 cm, 5 × 5 cm, 7 × 7 cm, 10 × 10 cm patch, respectively, (2) using different numbers of independent shunt dampers on the randomized thin plate and (3) using different patch configurations, i.e. PZT shunt dampers connected in series vs. parallel vs. independent.

The finite benchmark model consists of a simply supported thin plate attached with a PZT patch using properties shown in Table 1. Twenty point masses are distributed randomly (using rand function in matlab) within 90% from simply-supported boundaries of the thin plate to create randomness/structural uncertainties; where fifty ensembles (location of point masses are changed each time) are taken and then the responses are averaged out to get ensemble average. In the simulation, these point masses' locations are changed 50 times to get 50 ensembles.

**Table 1:** Properties of plate and PZT patch used.

Properties	Plate (AL 1100)	PZT patch (PZT-5H)
Young’s Modulus	80 × 10^9^ Pa	63 × 10^9^ Pa
Density	2710 kg/m^3^	7800 kg/m^3^
Length × width × thickness	0.8 × 0.6 × 0.0007 (m)	5 × 5 × 0.05 cm
Poisson’s ratio	0.33	0.31
Piezoelectric constant, d_31_ = d_32_	–	− 300 × 010^−12^ (V/m)

The subsequent investigations focused on frequency range where MOF > 2 lies in; this is where the non-deterministic response characteristics begin to exhibit for the thin plate. According to its frequency range, the MOF quantity is equated using Eq. (28) and found out to be > 190 Hz.

### Parametric study 1: effect of patch size

#### Effect of patch size to the estimation of effective line moment mobility, $Y_{\infty }^{eff}$

The following study investigates the effect of using different patch size, i.e. the length of line moment excitation on the thin plate; to the estimation of effective line moment mobility, $Y_{\infty }^{eff}$ derived in Eq. (27). A square patch with identical properties is used but with different side length for each case, i.e. 3 × 3 cm, 5 × 5 cm, 7 × 7 cm and 10 × 10 cm.

**Figure 6:**
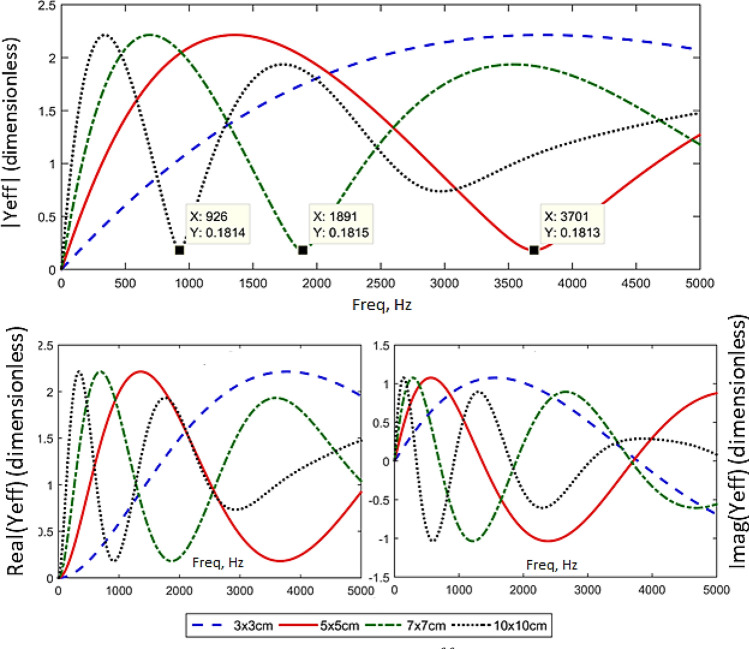
The estimation of effective line moment mobility, $Y_{\infty }^{eff}$ with varying PZT patch dimensions (graph in linear scale).

Figure 6 shows the effect of different patch size (length of line moment) to the estimation of effective line moment mobility of the non-deterministic thin plate, $Y_{\infty }^{eff}$. The plot highlights that, at a lower frequency up to about 500 Hz, the bigger is the patch actuator, the higher effective line moment mobility it produces. This effect becomes less important at higher frequencies, where dips beginning to occur earlier w.r.t frequency as patch size is increased. This effect is due to two factors. The first factor is the mass effect in which a bigger patch means an increase in weight; therefore, the mobility tends to roll off more efficiently for a bigger patch. The second factor is the ratio between the bending wavelength (Eq. (29)) and the patch actuator’s size. The smaller is the ratio between flexural wavelength and the patch size, the greater is the actuation effect. Up to frequencies where the patch's size equals an integer number of the bending wavelength, the magnitude of mobility rolls off to approach zero, this is where the cut-off frequency lies^27,28^.

29 $$\lambda = \frac{2\pi }{{k_{B} }} = \frac{2\pi }{{\sqrt[4]{{\omega^{2} \rho h/D}}}}.$$

**Figure 7:**
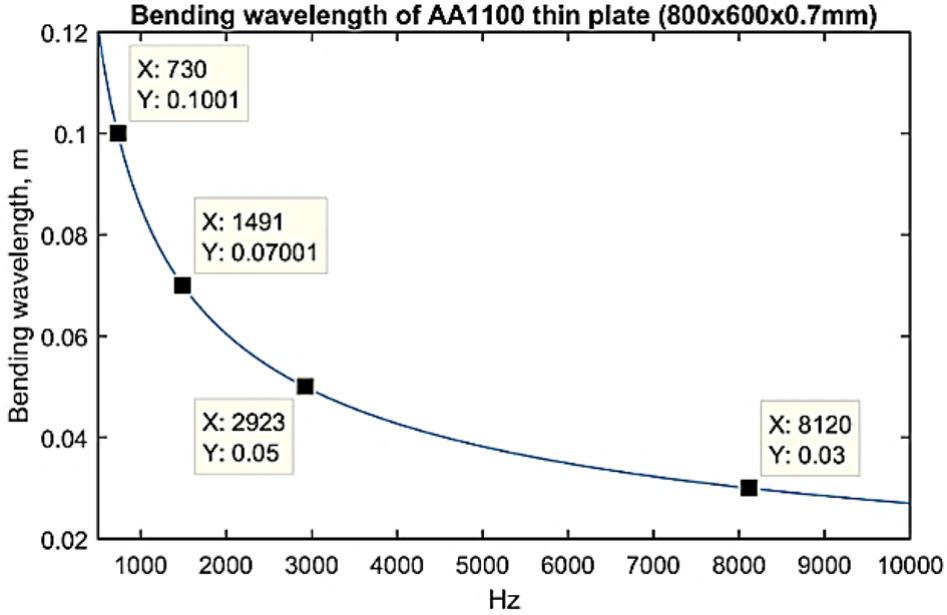
Bending wavelength for Aluminum plate 800 × 600 × 0.07 mm. The cut off frequencies for different patch size (3 cm, 5 cm, 7 cm and 10 cm) are determined.

Referring to Fig. 7, the cut-off frequencies for patch actuator of size 3 cm × 3 cm, 5 cm × 5 cm, 7 cm × 7 cm and 10 cm × 10 cm happened around 8120 Hz, 2923 Hz, 1491 Hz and 730 Hz, respectively. These frequencies serve as an estimation only due to the complexity of the resulting wave by the line moments in both *x* and *y* directions on the thin plate; therefore, the dips in Fig. 6 did not occur exactly at the theoretical cut-off frequencies as in Fig. 7. Also, note that these values are highly dependent on the material properties of the host structure.

#### Effect of patch size to the electrical shunt impedance, $Z_{sh}$

**Figure 8:**
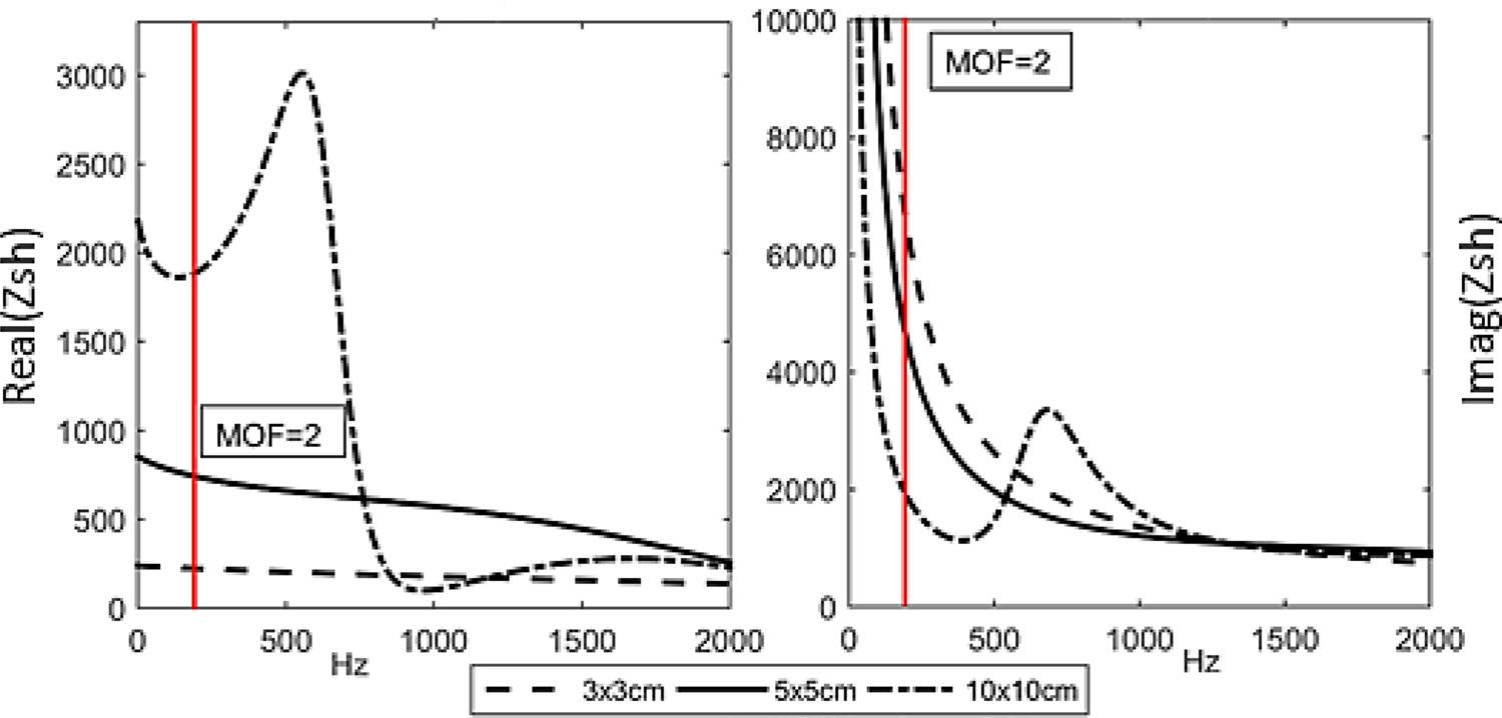
Real and imaginary values for the impedance of electrical shunt, $Z_{sh}$ using 3 × 3 cm, 5 × 5 cm and 10 × 10 cm patch. The red vertical line shows the frequency where MOF = 2.

Subsequently, the effect of changing patch size to the controller’s performance is desired. Figure 8 shows the real and imaginary curves of $Z_{sh}$ using relationship in Eq. (11) and the estimation of effective line moment mobility, $Y_{\infty }^{eff}$ as derived in Eq. (27) for different patch size. The real value of the shunt impedance is ideally the dissipative element in the circuit. It can be seen that all curves eventually level off to its smallest amplitude as the frequency is made higher. The occurrence of dips for a larger patch is due to the bending wavelength limitation that happened earlier than the smaller patch.

**Figure 9:**
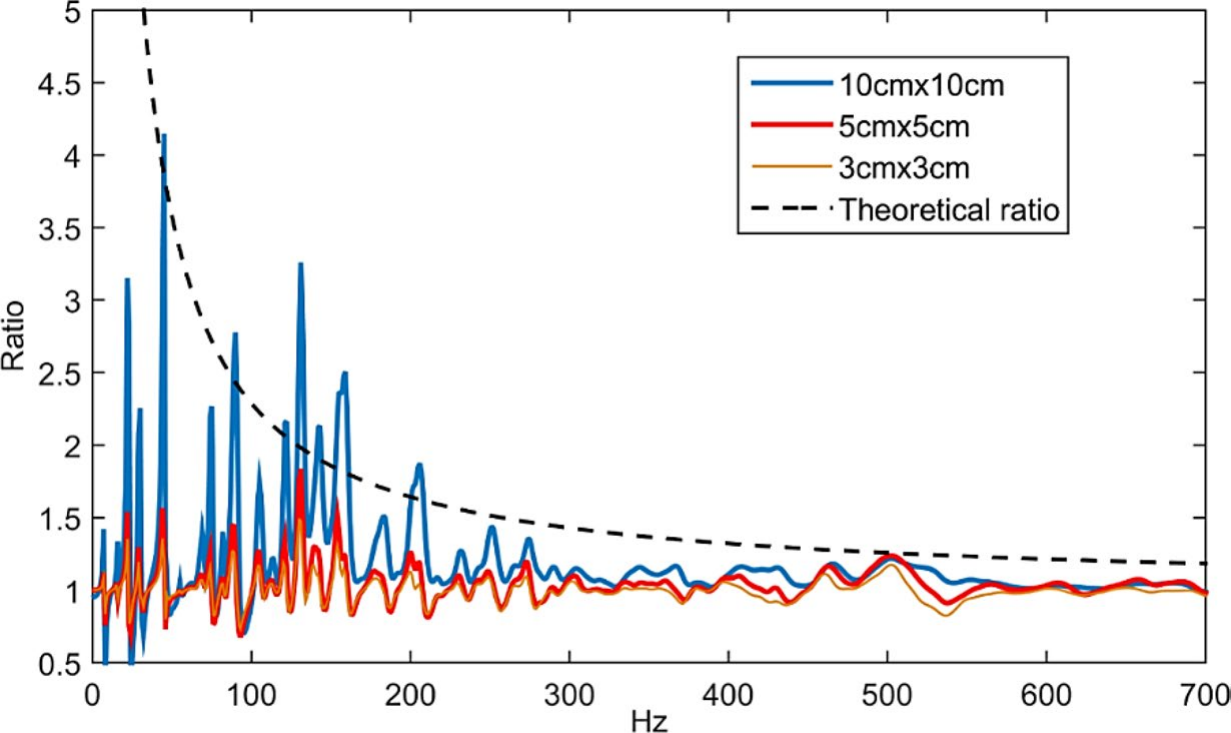
Uncontrolled/controlled plate energy when attached with optimal PZT shunt damper at different sizes, compared with theoretical energy ratio as derived in Eq. (21).

Figure 9 shows the effect of changing patch size on the shunt damping performance on the non-deterministic thin plate in terms of energy ratio as in Eqs. (20) and (6) for the theoretical curve. Relatively, the bigger the patch is, the better the shunt performance can be seen, essentially at lower frequency region (MOF < 2). Even though the larger patch has a higher value of the dissipative element, *Real(*$Z_{sh}$*)* as shown in Fig. 8, this does not significantly reduce the energy of the system at MOF > 2 since the structure consists of both real and imaginary impedance value. Therefore, at a much higher frequency, the shunt damping performance levels off to the theoretical curve, regardless of the patch size used. Furthermore, given that bigger patch size has a smaller cut-off frequency as demonstrated in Fig. 7, it is not always favorable to opt for a bigger patch for non-deterministic control using PZT patch.

From the simulation studies for different patch size, it can be deduced that for actuation and control of Non-DS using PZT patch, it is crucial to choose patch size that is small enough to have smaller ratio between bending wavelength and patch size for higher flexural actuation, and to avoid bending wavelength limitation; but large enough to have significant $Y_{\infty }^{eff}$ magnitude at the frequency range of interest (MOF > 2) of the non-deterministic substructure in question, for better control effectiveness.

### Parametric study 2: effect of the circuit configuration of piezoelectric shunt damper on the non-deterministic thin plate

**Figure 10:**
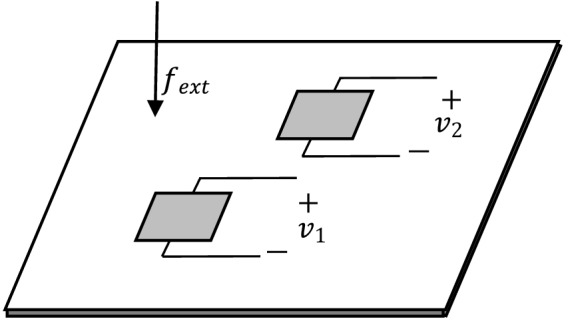
Schematic of two PZT patches attached on a thin plate with an external voltage applied at its terminals.

This subsection presents simulation studies on optimal PZT shunt dampers’ control performance on a Non-DS when the patch is connected in series and parallel, respectively. Two identical PZT patches are used in this study, as shown in Fig. 10, assuming all PZT properties are the same for all cases. For mathematical modelling, consider two identical PZT patches attached at a different location on a thin plate, the coupled electromechanical equations of the PZT elements can be written as follows^29–31^:

30 $$M_{1} \ddot{w} + K_{1} w - \Gamma_{1} v_{1} \left( t \right) = f_{ext} \left( t \right),$$

31 $$\Gamma_{1}^{T} w + C_{p1} v_{1} \left( t \right) = q_{1} \left( t \right),$$

32 $$M_{2} \ddot{w} + K_{2} w - \Gamma_{2} v_{2} \left( t \right) = f_{ext} \left( t \right),$$

33 $$\Gamma_{2}^{T} w + C_{p2} v_{2} \left( t \right) = q_{2} \left( t \right).$$

The total equation of motion for the thin plate attached with PZT patches and when both are connected to an external voltage (actuator mode) is:

34 $$M_{tot} \ddot{w} + K_{tot} w - \Gamma_{1} v_{1} \left( t \right) - \Gamma_{2} v_{2} \left( t \right) = f_{ext} \left( t \right),$$

where $M_{tot}$ is the total mass of the system (sum of $M_{plate} , M_{1}$ and $M_{2}$), $K_{tot}$ is the total effective stiffness of the system (sum of $K_{plate} \left( {1 + j\eta } \right)$, ${\text{K}}_{1}$ and $K_{2}$) and $f_{ext}$ is an external force applied to the thin plate, $\Gamma_{k}$ is the electromechanical coupling for the *k*th patch, *w* is displacement and $v_{k}$ is the voltage applied across the terminal for patch *k.* Subscripts 1 and 2 denote PZT patch 1 and 2, respectively.

#### Piezoelectric patches in parallel configuration

**Figure 11:**
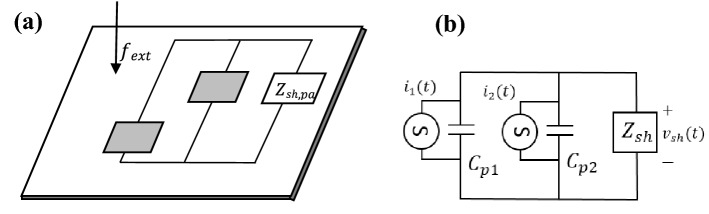
(**a**) The two piezoelectric shunt dampers in the parallel configuration on a thin plate and (**b**) its equivalent electrical circuit representation with shunt circuit, $Z_{sh,pa}$.

For the first case, consider the two PZT patches are connected in parallel with one shunt circuit also connected in parallel as depicted in the following Fig. 11a,b:

For a thin plate attached with two PZT patches connected in parallel, the electrical boundary conditions of the system can be written as:

35 $$v_{1} \left( t \right) = v_{2} \left( t \right) = v_{sh} \left( t \right) = v\left( t \right),$$

36 $$q_{sh} \left( t \right) = q_{1} \left( t \right) + q_{2} \left( t \right),$$

where,

$$\begin{aligned} v_{sh} \left( t \right) = & - \dot{q}_{sh} \left( t \right)Z_{sh,pa} \\ = & - (\dot{q}_{1} + \dot{q}_{2} )Z_{sh,pa} , \\ \end{aligned}$$

$$V_{sh} \left( s \right) = - \left( {sV_{1} C_{p1} + s\Gamma_{1}^{T} W_{pa} + sV_{2} C_{p2} + s\Gamma_{2}^{T} W_{pa} } \right)Z_{sh,pa} ,$$

$$V_{pa} \left( {1 + sC_{p1} Z_{sh,pa} + sC_{p2} Z_{sh,pa} } \right) = - s\left( {\Gamma_{1}^{T} + \Gamma_{2}^{T} } \right)W_{pa} Z_{sh,pa} ,$$

37 $$V_{pa} = \frac{{ - s\left( {\Gamma_{1}^{T} + \Gamma_{2}^{T} } \right)W_{pa} }}{{\left( {1 + sC_{p1} Z_{sh,pa} + sC_{p2} Z_{sh,pa} } \right)}}.$$

The terms $q_{k}$, $v_{k}$, are the charge and voltage at the *k*th branch, respectively. The voltage, $V_{pa}$ in Eq. (36) can be written as such since the voltage at each branch is the same for the parallel case. Therefore, the energy for the thin plate attached with optimal parallel shunt dampers can be deduced to be:

38 $$E_{C,pa} = \frac{1}{2}W_{C,pa}^{T} K_{plate} W_{C,pa} ,$$

39 $$W_{C,pa} = \left[ {s^{2} M_{tot} + K_{tot} + \frac{{s(\Gamma_{1} + \Gamma_{2} )\left( {\Gamma_{1}^{T} + \Gamma_{2}^{T} } \right)Z_{sh,pa} }}{{1 + sZ_{sh,pa} \left( {C_{p1} + C_{p2} } \right)}}} \right]^{ - 1} *F_{ext} \left( s \right).$$

The shunt damping circuit, $Z_{sh,pa}$ in this case needs to be the complex conjugate of the impedance seen from the circuit’s point of view, which is the complex conjugate of the electrical-equivalent impedance of the host structure parallel with the impedance of the piezoelectric patch, $X_{Cp,k}$ for every *k*th patch connection, where all patches are connected in parallel. For clarity, $Z_{sh,pa}$ can be written mathematically using the following relationship:

40 $$Z_{sh,pa} = conj[(\overline{Z}_{\infty ,1} ||Z_{Cp,1} )||(\overline{Z}_{\infty ,2} ||Z_{Cp,2} )].$$

**Figure 12:**
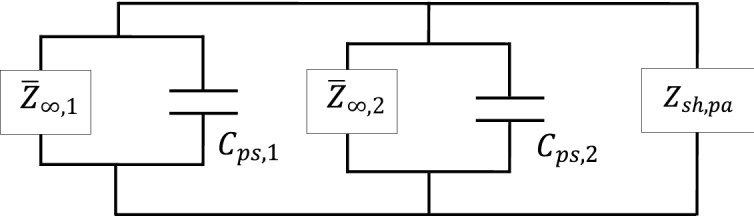
Equivalent electrical impedance representation of PZT shunt dampers connected in parallel.

Illustratively, Eq. (39) can be represented as in Fig. 12:

Recall that the term $\overline{Z}_{\infty ,k}$ in Eq. (12) is the equivalent electrical impedance converted from its mechanical impedance ‘faced’ by the non-deterministic thin plate when being subjected by the excitation of the *k*th PZT patch actuator, $Z_{\infty ,k}$.

#### Piezoelectric patches in a series configuration

**Figure 13:**
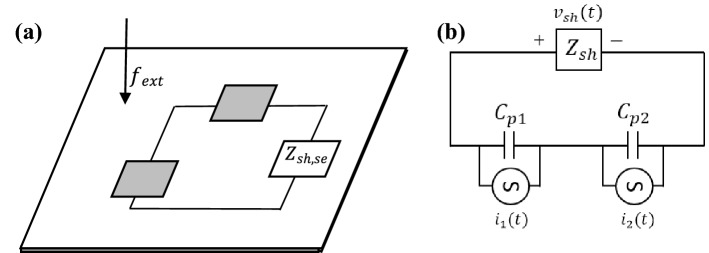
(**a**) The two piezoelectric shunt dampers in a series configuration on thin-plate (**b**) its equivalent electrical circuit representation with shunt circuit, $Z_{sh,se}$.

For the second case, consider the two PZT patches are connected in series as shown in Fig. 13a,b, and with one shunt circuit.

For a thin plate attached with two PZT patches connected in series, the electrical boundary conditions of the system can be written as follows:

41 $$V_{sh} \left( t \right) = V_{1} \left( t \right) + V_{2} \left( t \right),$$

42 $$q_{sh} \left( t \right) = q_{1} \left( t \right) = q_{2} \left( t \right) = q\left( t \right),$$

where:

43 $$V_{sh} \left( t \right) = - \dot{q}_{sh} \left( t \right)Z_{sh,se} ,$$

44 $$\frac{{q_{1} }}{{C_{p1} }} - \frac{{\Gamma_{1}^{T} w}}{{C_{p1} }} + \frac{{q_{2} }}{{C_{p2} }} - \frac{{\Gamma_{2}^{T} w}}{{C_{p2} }} = - \dot{q}_{sh} Z_{sh,se} ,$$

45 $$Q_{se} \left( {\frac{1}{{C_{p1} }} + \frac{1}{{C_{p2} }}} \right) - W_{se} \left( {\frac{{\Gamma_{1}^{T} }}{{C_{p1} }} + \frac{{\Gamma_{2}^{T} }}{{C_{p2} }}} \right) = - sQ_{se} Z_{sh,se} ,$$

46 $$Q_{se} = \frac{{\left( {\frac{{\Gamma_{1}^{T} }}{{C_{p1} }} + \frac{{\Gamma_{2}^{T} }}{{C_{p2} }}} \right)W_{se} }}{{\left( {\frac{1}{{C_{p1} }} + \frac{1}{{C_{p2} }}} \right) + sZ_{sh,se} }}.$$

The charge, $Q_{se}$ in Eq. (45) can be written as such since the charge $q_{k}$ between $C_{pk}$ is the same when connected in series. After some algebraic manipulation, the energy for a thin plate attached with optimal series shunt dampers can be deduced to be:

47 $$E_{C,se} = \frac{1}{2}W_{C,se}^{T} K_{plate} W_{C,se} ,$$

48 $$W_{C,se} = \left[ {s^{2} M_{tot} + K_{tot} + \frac{{\frac{{(\Gamma_{1} - \Gamma_{2} )(\Gamma_{1} - \Gamma_{2} )^{T} }}{{\left( {C_{p1} C_{p2} } \right)}} + \left( {\frac{{\Gamma_{1} \Gamma_{1}^{T} }}{{C_{p1} }} + \frac{{\Gamma_{2} \Gamma_{2}^{T} }}{{C_{p2} }}} \right)sZ_{sh,se} }}{{1 + sZ_{sh,se} \left( {\frac{{C_{p1} C_{p2} }}{{C_{p1} + C_{p2} }}} \right)}}} \right]^{ - 1} \times F_{ext} \left( s \right).$$

The shunt damping circuit, $Z_{sh,se}$ in this particular case needs to be the complex conjugate of the electrical-equivalent impedance of the host structure parallel with the impedance of the piezoelectric patch, $X_{Cp,k}$ for every *k*th patch connection, where all patches are connected in series. For clarity, the following mathematical representation can be written:

49 $$Z_{sh,se} = conj[(\overline{Z}_{\infty ,1} ||Z_{Cp,1} ) + (\overline{Z}_{\infty ,2} ||Z_{Cp,2} )].$$

**Figure 14:**
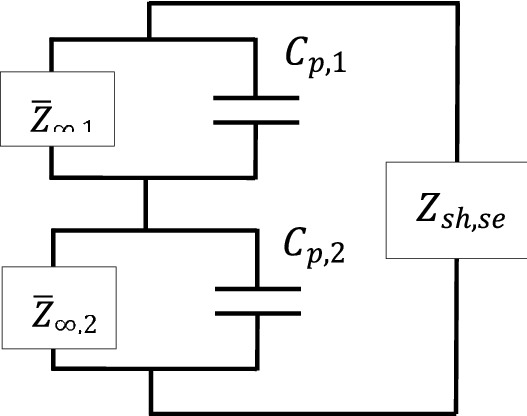
Equivalent electrical impedance representation of PZT shunt dampers in series.

Illustratively Eq. (48) can be depicted as in Fig. 14.

Similarly like the parallel case, the term $\overline{Z}_{\infty ,k}$ in Eq. (48) is the equivalent electrical impedance converted from mechanical impedance ‘faced’ by the non-deterministic thin plate when being subjected by the excitation of the *k*th PZT patch actuator, $Z_{\infty ,k}$, at connection *k.*

#### Energy reduction ratio of series and parallel compared with independent PZT shunt dampers

**Figure 15:**
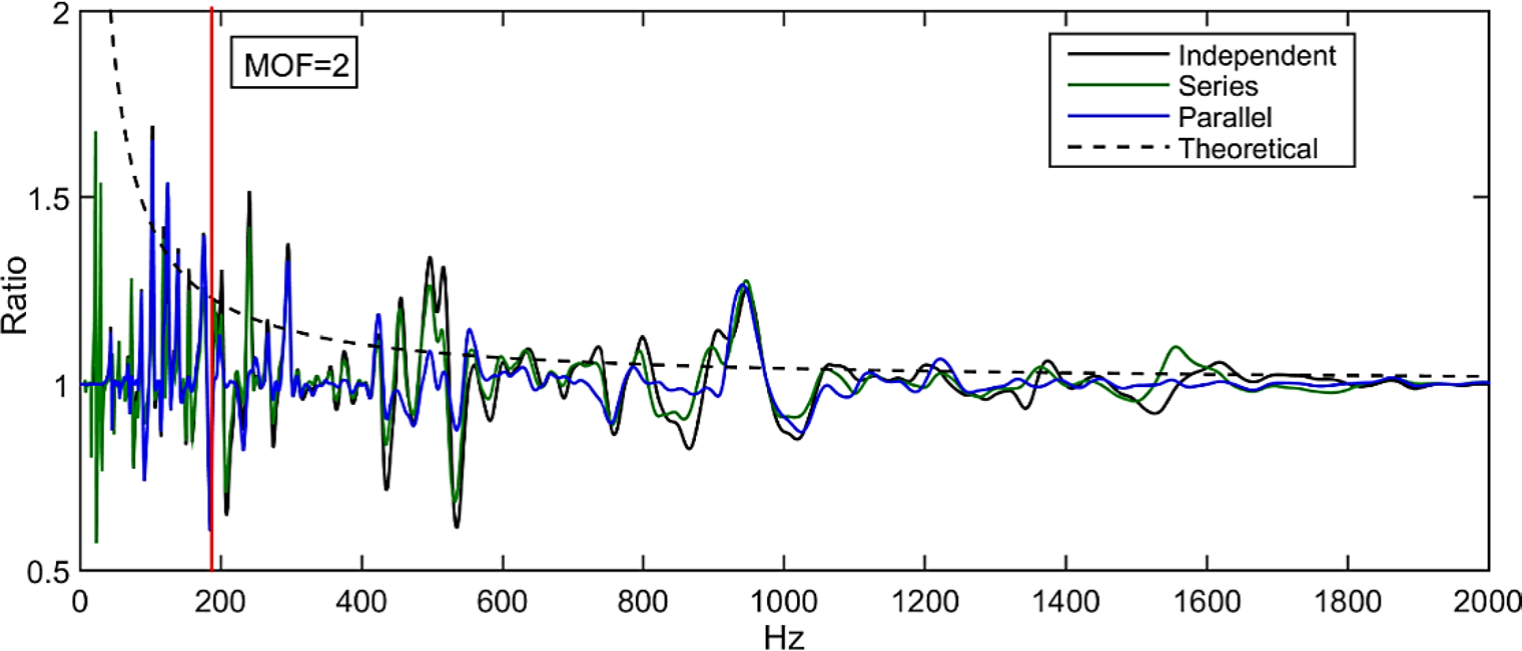
Controlled/uncontrolled plate energy when attached with optimal PZT shunt damper at different configurations, compared with theoretical energy ratio as derived in Eq. (21).

The control performance of the PZT shunt dampers connected in series and parallel as derived previously will be compared with independent shunt dampers along with the theoretical energy reduction curve, $E_{ratio, Inf}$ (Eq. (6)) for comparison. Figure 15 compares the energy reduction ratio curve by using two PZT shunt dampers on the non-deterministic thin plate connected in different configurations; series, parallel and independent, respectively. From the simulation results, at earlier frequencies, patches connected in series exhibits better energy reduction followed by independent and parallel connection with about the same magnitude. At higher frequency range where MOF > 2 lies in, the optimal PZT shunt dampers connected in series and independent seem to lead, specifically at frequency 400-600 Hz and 900 Hz. However, all configurations eventually level off to the theoretical curve at a much higher frequency range, signifying the maximum achievable energy reduction.

From this analysis, since there is no distinctive difference that can be seen in terms of the control performance especially where MOF > 2, the circuit impedance $Z_{sh}$ can be designed by using any of the three patch configurations. This means that there can be more variety of shunt circuit designs using *RLC* components in various combinations to replicate the theoretical impedance in Eq. (11). However, considering the complexity of the circuit needed to be designed as illustrated in Eqs. (39) and (48) when more patch is used, it is deemed appropriate to use an independent connection to achieve simplicity and practicality.

### Parametric study 3: effect of number of distributive PZT shunt damper for energy dissipation of the non-deterministic thin plate

The following study investigates the effect of using a different number of independent PZT shunt dampers for controlling the non-deterministic plate. Identical PZT shunt dampers are used for this study (5 cm × 5 cm) distributed evenly across the randomized thin plate.

**Figure 16:**
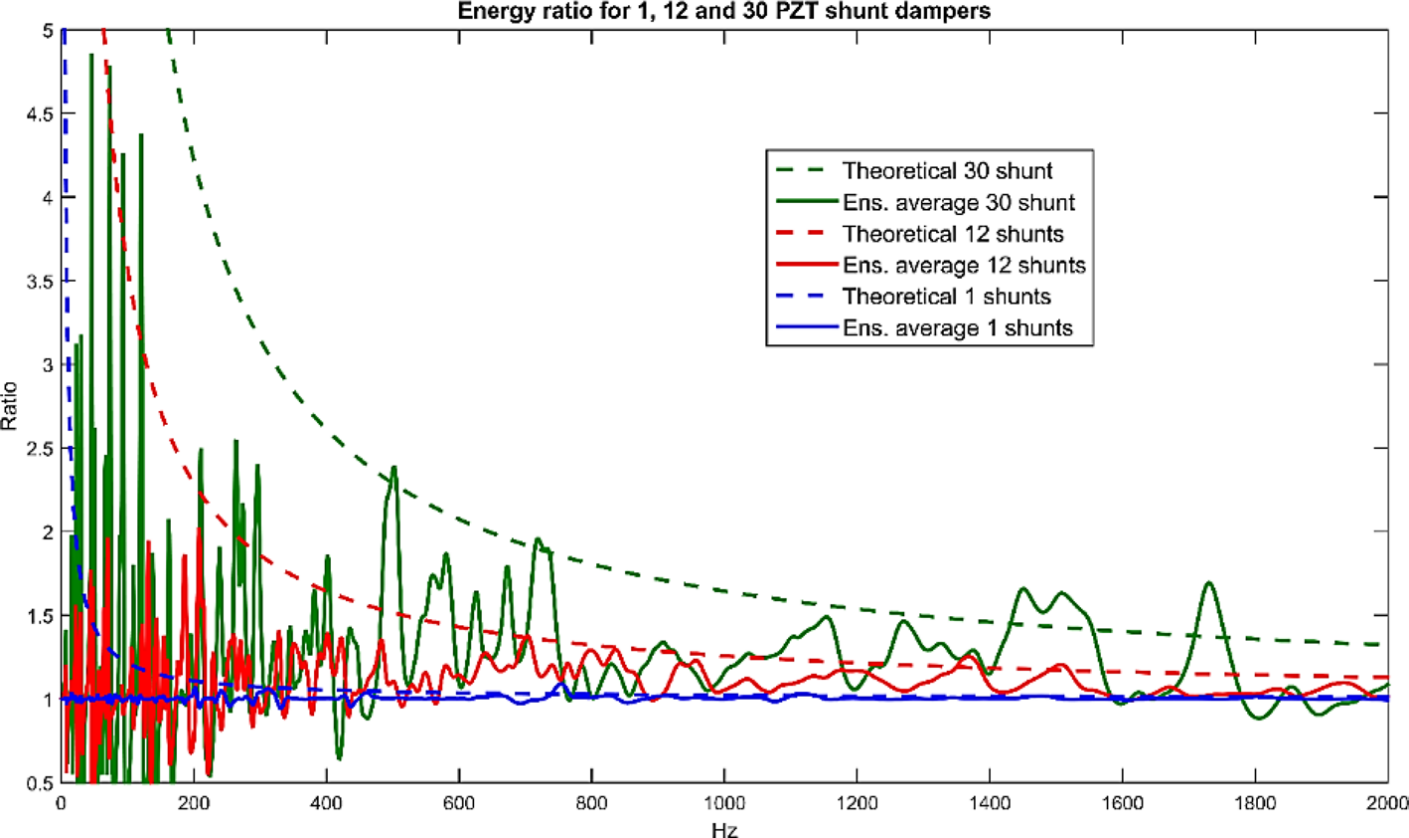
Controlled/uncontrolled plate energy when attached with various quantity of optimal PZT shunt dampers, compared with theoretical energy ratio as derived in Eq. (21).

Figure 16 shows the energy reduction ratio when the non-deterministic plate is attached with 1, 6, 9, 12, 16 and 30 optimal PZT shunt dampers, respectively. The finite model’s ensemble average seems to agree well with the theoretical curve for respective cases as shown in the figure, especially at MOF > 2 range (> 190 Hz). The simulation shows that better energy reduction can be achieved when using more PZT shunt dampers on the non-deterministic thin plate. However, more patch means adding more weight and stiffness to the structure, which is not usually favorable. Therefore, the number of PZT patch used (or generally, number of controllers used) needs to be compensated with the desired control effect of the Non-DS.

## Conclusions

From this research, the real and imaginary expressions of $Z_{\infty ,k}$, that is the impedance ‘faced’ by the non-deterministic thin plate when being subjected by the excitation of line moments in rectangular/square shape distribution is derived using double integration of infinite mobility resulting to *effective line moment mobility* and eventually used in the expression of shunt circuit impedance, $Z_{sh,k}$. Parametric investigations showed that by using more PZT shunt dampers, better energy reduction of the Non-DS could be achieved. Also, larger patch produced better energy reduction but by keeping in mind that larger patch means bending wavelength limitation will occur earlier. Also, by using more patches with bigger size means more weight is added to the system, which is not desirable. Therefore, the quantity and size of the patch used on the Non-DS need to be compensated with the control performance. Also, no conclusive difference can be seen for energy reduction of the plate when the patch is connected in series, parallel or independently. Therefore, the shunt circuit can be designed in either configuration in the way that is more convenient. However, considering the complexity of the circuit needed to be designed as illustrated in Eqs. (39) and (48) when more patches are used, independent PZT shunt damper design where each patch is connected to its own shunt circuit is deemed more practical. Last but not least, it is important to note that finite energy reduction ratio curves fall under the theoretical curve (Eq. (6)) for every case, which means the curve serves as an envelope for the highest energy dissipation attainable from a Non-DS when directly attached with optimal controllers.
